# Reversal of CYLD phosphorylation as a novel therapeutic approach for adult T-cell leukemia/lymphoma (ATLL)

**DOI:** 10.1038/s41419-020-2294-6

**Published:** 2020-02-05

**Authors:** Xin Xu, Matko Kalac, Michael Markson, Mark Chan, Joshua D. Brody, Govind Bhagat, Rosalind L. Ang, Diana Legarda, Scott J. Justus, Feng Liu, Qingshan Li, Huabao Xiong, Adrian T. Ting

**Affiliations:** 1 Precision Immunology Institute, Icahn School of Medicine at Mount Sinai, New York, NY 10029 USA; 2 Department of Geriatrics, Hematology & Oncology Ward, Guangzhou First People’s Hospital, School of Medicine, South China University of Technology, Guangzhou, GuangDong 510180 People’s Republic of China; 3 Tisch Cancer Institute, Icahn School of Medicine at Mount Sinai, New York, NY 10029 USA; 4 Department of Pathology and Cell Biology, Columbia University Medical Center, New York Presbyterian Hospital, New York, NY 10032 USA; 5 Department of Hematology, Guangzhou First People’s Hospital, School of Medicine, South China University of Technology, GuangDong, 510180 People’s Republic of China; 6 Department of Immunology, Mayo Clinic, Rochester, MN 55905 USA

**Keywords:** T-cell lymphoma, Tumour-suppressor proteins, Apoptosis, Ubiquitylation

## Abstract

Adult T-cell leukemia/lymphoma (ATLL) is a malignancy of mature T cells associated with chronic infection by human T-cell lymphotropic virus type-1 (HTLV-1). ATLL patients with aggressive subtypes have dismal outcomes. We demonstrate that ATLL cells co-opt an early checkpoint within the tumor necrosis factor receptor 1 (TNFR1) pathway, resulting in survival advantage. This early checkpoint revolves around an interaction between the deubiquitinase CYLD and its target RIPK1. The status of RIPK1 K63-ubiquitination determines cell fate by creating either a prosurvival signal (ubiquitinated RIPK1) or a death signal (deubiquitinated RIPK1). In primary ATLL samples and in cell line models, an increased baseline level of CYLD phosphorylation was observed. We therefore tested the hypothesis that this modification of CYLD, which has been reported to inhibit its deubiquitinating function, leads to increased RIPK1 ubiquitination and thus provides a prosurvival signal to ATLL cells. CYLD phosphorylation can be pharmacologically reversed by IKK inhibitors, specifically by TBK1/IKKε and IKKβ inhibitors (MRT67307 and TPCA). Both of the IKK sub-families can phosphorylate CYLD, and the combination of MRT67307 and TPCA have a marked effect in reducing CYLD phosphorylation and triggering cell death. ATLL cells overexpressing a kinase-inactive TBK1 (TBK1-K38A) demonstrate lower CYLD phosphorylation and subsequently reduced proliferation. IKK blockade reactivates CYLD, as evidenced by the reduction in RIPK1 ubiquitination, which leads to the association of RIPK1 with the death-inducing signaling complex (DISC) to trigger cell death. In the absence of CYLD, RIPK1 ubiquitination remains elevated following IKK blockade and it does not associate with the DISC. SMAC mimetics can similarly disrupt CYLD phosphorylation and lead to ATLL cell death through reduction of RIPK1 ubiquitination, which is CYLD dependent. These results identify CYLD as a crucial regulator of ATLL survival and point to its role as a potential novel target for pharmacologic modification in this disease.

## Introduction

Adult T-cell leukemia/lymphoma (ATLL) is a malignancy of mature T lymphocytes driven by human T-cell lymphotropic virus type-1 (HTLV-1). This disease is particularly prevalent in certain endemic populations^1^. Infection with HTLV-1 occurs in infancy, and an estimated 5% of the infected population will develop ATLL after a decades-long period of latency^2–4^. The multistep oncogenic process begins with integration of the virus into the host genome, after which the viral transactivator protein Tax interacts with various pathways important for cell survival and apoptosis^5–10^. In addition, immune evasion of the oligoclonal cells, secondary epigenetic changes, and mutations in the T-cell receptor pathway result in clonal proliferation that clinically manifests as one of four disease subtypes: acute, lymphomatous, chronic, and smoldering^6,11–13^. Despite intensive chemotherapy, the prognosis for the aggressive subtypes (acute and lymphomatous) is abysmal with a median survival measured in months. This translates into 4-year overall survival rates of only 11–16%^14^. Particularly poor outcomes have been described for the Caribbean populations that comprise a significant ethnic group of ATLL patients in the United States^15^. Although effective for long term disease control, allogeneic stem cell transplant is reserved for those patients with good performance status, but this modality is associated with high transplant related mortality rates^16–18^. Promising novel treatments for ATLL including antiviral therapy^19,20^ and use of a monoclonal antibody (mogamulizumab)^21,22^ have low complete response rates. Similarly disappointing results were seen with a PD1 inhibitor, with rapid disease progression noted shortly following nivolumab administration^23^. New treatment options are thus urgently needed^24^ that are based on a better understanding of mechanisms regulating ATLL tumor cell survival.

One of the best-characterized pathways that determines cellular fate is the tumor necrosis factor receptor 1 (TNFR1) pathway. Binding of TNF to its receptor induces a survival response in the vast majority of primary and transformed cells, but it can also trigger a death response if cell death checkpoints are disrupted. It has been proposed that there are two such checkpoints in the TNFR1 pathway—an early transcription-independent checkpoint and a late NFκB-dependent checkpoint^25–27^. Because these checkpoints function to antagonize the cell death machinery, there is a high likelihood that they can be co-opted by tumors to maintain cellular survival. Indeed, NFκB/Rel family members are proto-oncogenes and the role of the NFκB-dependent checkpoint in tumorigenesis, via its induction of anti-cell death genes, is now well established. In contrast, the role of the early NFκB-independent cell death checkpoint in tumor biology is not well understood. This early checkpoint is centered on the TNFR1 signaling molecule RIPK1, which has a bifunctional role where it can signal to induce either cell survival or cell death. A critical insight came when it was discovered that non-degradative lysine 63 (K63)-linked poly-ubiquitination of RIPK1 functions as a toggle that regulates whether RIPK1 generates a survival signal (when it is ubiquitinated) or a death signal (when ubiquitination is blocked)^28^. Therefore, non-degradative ubiquitination of RIPK1 serves a prosurvival function by suppressing the proclivity of RIPK1 to engage the cell death pathway^26,29^. Because this early checkpoint is regulated by ubiquitination of RIPK1, ubiquitin E3 ligases and deubiquitinases are critical regulators of this checkpoint and potentially, tumor cell survival. Consistent with this notion, SMAC mimetics have been shown to induce RIPK1-dependent death of tumor cells^30–32^. These compounds disrupt the early checkpoint by degrading inhibitor of apoptosis proteins (IAPs), which are E3 ligases that catalyze K63-linked ubiquitination of RIPK1^30,32^, thereby presumably reducing these modifications of RIPK. In addition to ubiquitination, the death-signaling function of RIPK1 is also inhibited by phosphorylation. Two classes of kinases, the IKK family and MK2, have been shown to be involved^33–40^.

While the IAPs catalyze the conjugation of K63-linked ubiquitin chains, the removal of these chains is mediated by the CYLD deubiquitinase, which targets RIPK1 and other molecules in the TNFR1 signaling pathway^41–43^. CYLD is a tumor suppressor that was initially identified in familial tumors including cylindromatosis, familial trichoepithelioma and Brooke–Spiegler syndrome. It has also been implicated in hematological cancers and it is thought to function as a tumor suppressor, largely by inhibiting NFκB signaling^44^. CYLD has been implicated in the regulation of the early TNFR1 checkpoint^29^ and it was reported to be required for the induction of RIPK1-dependent apoptosis and necroptosis by TNF^25,32,45–47^. CYLD removes K63-linked ubiquitin chains from RIPK1 and converts RIPK1 to a death-signaling molecule^25,47^. Therefore, it is possible that another tumor suppressor function for CYLD may be to activate the RIPK1 death-signaling response, and antagonizing this death pathway by deleting or otherwise inhibiting CYLD would be expected to provide a survival advantage to tumors. Previous studies have reported that CYLD deubiquitinating activity can be inhibited by phosphorylation^48,49^. In addition, HTLV-1 TAX is found in a complex with CYLD and CYLD is constitutively phosphorylated in HLTV-1 transformed T cells^50^. We now report that phosphorylation of CYLD by IKK family kinases in HTLV-1 transformed T cells inhibits RIPK1 from activating the cell death pathway and inhibiting these kinases reactivates CYLD and RIPK1-dependent tumor cell death. Importantly, increased CYLD phosphorylation was found in primary ATLL patient samples.

## Results

### Increased CYLD phosphorylation is a frequent event in ATLL

**Fig. 1:**
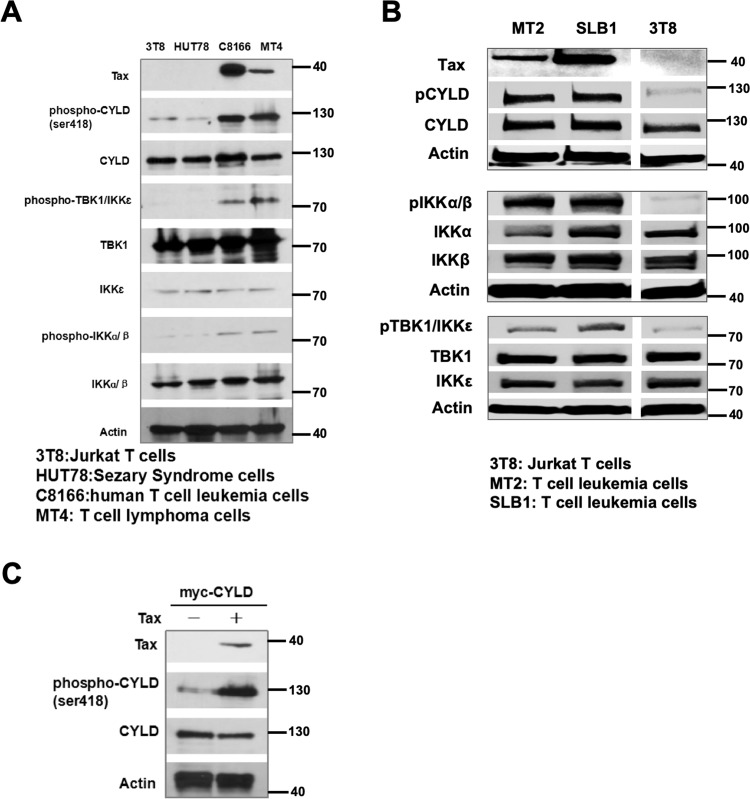
Increased CYLD phosphorylation is a frequent event in ATLL cells and is mediated by viral TAX oncoprotein. **a** Lysates from 3T8, HUT78, C1866, and MT4 cells were analyzed by blotting with the indicated antibodies. β-actin was blotted as a loading control. 3T8 is a Jurkat clone used as a negative control. HUT78 is a Sézary Syndrome cell line. C1866 and MT4 are HTLV-1-positive ATLL cell lines. **b** Lysates from 3T8, SLB1, and MT2 cells were analyzed by blotting with the indicated antibodies. β-actin was blotted as a loading control. 3T8 is a Jurkat clone used as a negative control. SLB1 and MT2 are HLTV-1-positive ATLL cell lines. **c** HEK293 EBNA cells were transfected with plasmids encoding a control protein or TAX together with that for myc-CYLD. Forty-eight hour post transfection, lysates were blotted for TAX, phospho-CYLD and CYLD.

We were prompted to examine the role of CYLD phosphorylation in ATLL lymphomagenesis based on reports that NEMO/IKKβ and IKKε can phosphorylate a cluster of serines between residues 418 and 444 of CYLD resulting in the inhibition of CYLD’s catalytic activity^48,49^. Since there are only a few reports of chromosomal loss or genetic inactivation of *CYLD* in human lymphomas^51^, and none reported in ATLL, we hypothesize that CYLD may be posttranslationally suppressed in these malignancies. We first analyzed CYLD phosphorylation in C8166 and MT4 T cell lines, which are HTLV-1-transformed T cells. Consistent with an earlier report^50^, western blotting with an antibody that detects phosphorylation of CYLD at serine 418 showed this posttranslational modification to be elevated in the HTLV-1-transformed cell lines (Fig. 1a). In addition, two more Tax positive cell lines (MT2 and SLB1) showed increased levels of CYLD phosphorylation (Fig. 1b). In all our experiments, we used lysates from Jurkat T cells (clone 3T8)^52^ as the negative control because of this cell line’s low basal levels of CYLD phosphorylation. We also confirmed that the antibody that detects phospho-S418 of CYLD is specific by using it to blot lysates taken from MT4 cells that were transduced with a control shRNA or a CYLD-targeting shRNA to generate CYLD-deficient cells (Supplementary Fig. 1). An immunoreactive band was detected by the phospho-S418 antibody in CYLD-sufficient cells but not CYLD-deficient MT4 cells.

Multiple members of the IKK family, including IKKβ, TBK1, and IKKε can phosphorylate CYLD^48,49,53,54^; hence we examined the activation status of these kinases. In all cases, we detected elevated phospho-TBK1/IKKε (serine 172) and phospho-IKKα/β (serines 176 and 180) (Fig. 1a, b). Due to amino acid homology between TBK1 and IKKε around serine 172, the phospho-specific antibody could not distinguish between phosphorylated TBK1 and IKKε. Likewise, the phospho-IKKα/β antibody is unable to distinguish between the two closely related kinases. Nonetheless, both subfamilies of IKK, which are known CYLD kinases^48,49,53^, are activated in all TAX-positive ATLL cells. Finally, we examined the phosphorylation status of CYLD in lysates of human ATLL cryo-preserved samples from which we were able to obtain sufficient protein to resolve by sodium dodecyl sulfate polyacrylamide gel electrophoresis (SDS-PAGE) for CYLD phosphorylation. In both samples, CYLD phosphorylation was elevated concomitant with that of TBK1/IKKε and IKKα/β (Supplementary Fig. 2). These results demonstrate that CYLD phosphorylation is elevated in human ATLL. HTLV-1 encodes the 40 kD oncogene TAX, which plays a key role in T-cell transformation^55,56^. We reasoned that since TAX is known to activate IKK and can associate with CYLD^50^, the TAX protein may be sufficient to induce CYLD phosphorylation. Transfection of a TAX-encoding plasmid into HEK293 EBNA cells confirmed that TAX by itself is sufficient to induce CYLD phosphorylation (Fig. 1c).

### IKK family kinases phosphorylate CYLD in the ATLL MT4 cell line

**Fig. 2:**
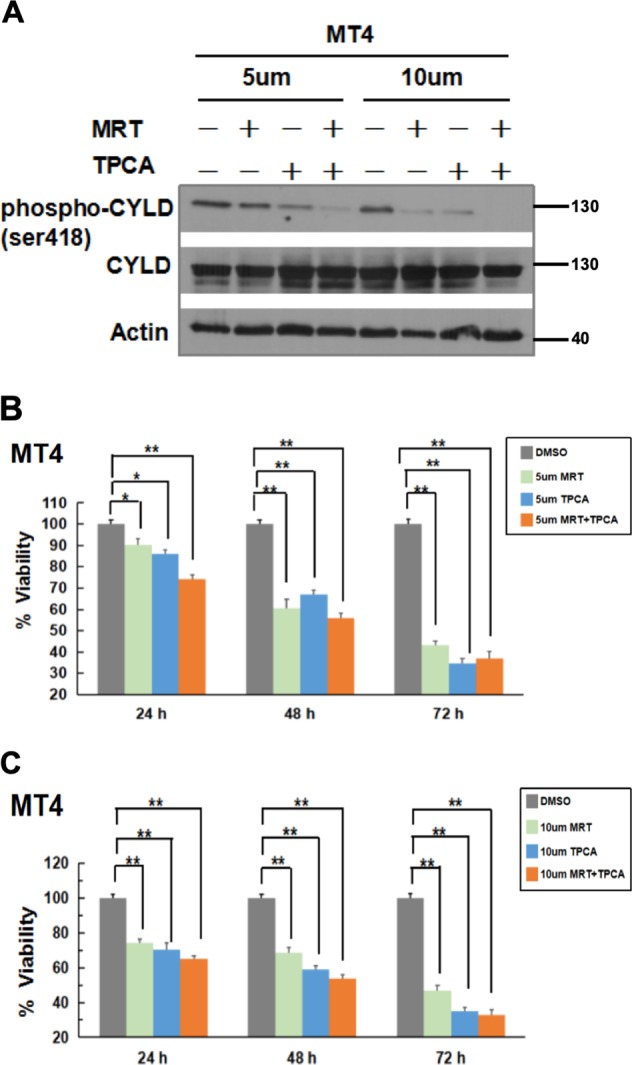
CYLD phosphorylation is mediated by IKKs and can be reversed by IKK inhibitors. **a** MT4 cells were treated with the indicated concentrations of MRT67307, TPCA, or both inhibitors for 24 h. DMSO was used as a negative control. Lysates were sequentially blotted with the indicated antibodies. **b**, **c** MT4 cells were treated as indicated with 5 µM (**b**) or 10 µM (**c**) of MRT67307, TPCA, or both inhibitors for 24–72 h. Cell viability was measured using the CellTiter-Glo assay for ATP levels. The viability of cells treated with DMSO was set at 100% for each time point. The results represent the mean ± S.D. from three independent experiments. *p < 0.05; **p < 0.01.

**Fig. 3:**
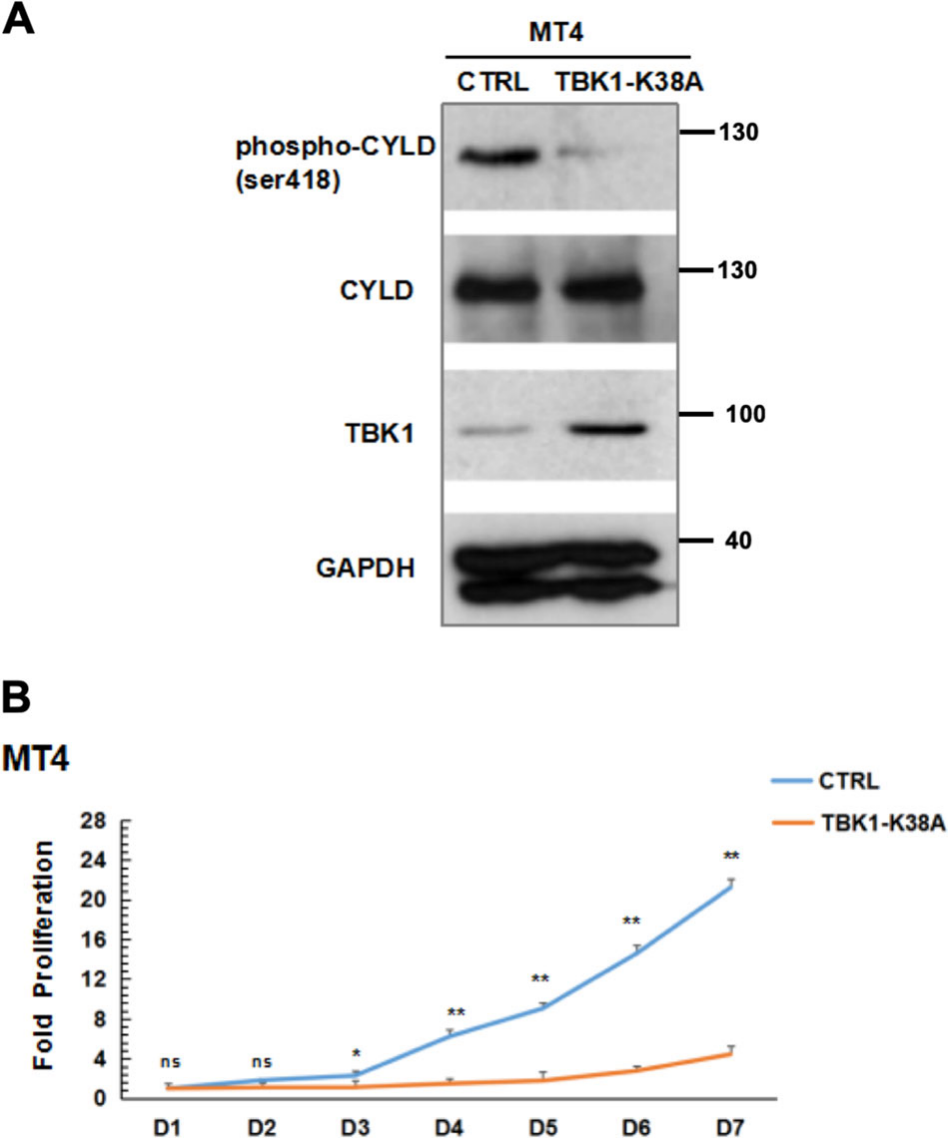
Kinase-inactive TBK1-K38A mutant decreases CYLD phosphorylation and inhibits proliferation. **a** MT4 cells were transduced with retroviruses encoding a control gene or a kinase-inactive TBK1-K38A mutant. After stable selection with puromycin for 3 weeks, lysates from the transfectants were blotted for phospho-CYLD, CYLD, TBK1, and GAPDH. **b** The two cell lines in (**a**) were seeded at 2.5 × 104 cells/well in multiple wells in 96-well plates. Triplicate wells from each cell line were harvested at 24-h interval and cellular proliferation analyzed by CellTiter-Glo. Each data point is the mean ± S.D. from three independent experiments. The ATP level at the initiation of the experiment (Day 1) for each cell line was set at 1 and those on subsequent days normalized to that value. ns no statistical difference; *p < 0.05; **p < 0.01.

To investigate further if IKK kinases are involved in phosphorylating CYLD in MT4 cells, we examined the effect of MRT67307 (TBK1/IKKɛ inhibitor) and TPCA (IKKβ inhibitor). At the two concentrations of 5 and 10 μM tested, both inhibitors reduced CYLD phosphorylation individually but the combination of these inhibitors was more potent in reducing CYLD phosphorylation (Fig. 2a). This suggests that both subfamilies of IKK can phosphorylate CYLD and that there is redundancy amongst the kinases in TAX-expressing cells. We next examined the impact on cell survival when CYLD phosphorylation was blocked by the IKK inhibitors following incubation with 5 or 10 μM of the two IKK inhibitors for 24–72 h. Individual IKK inhibitors led to MT4 cell death and combining the two led to a modest enhancement of cell death (Fig. 2b, c). Given the multiple kinases that phosphorylate CYLD and the potential redundancy amongst them as suggested by the inhibitor studies, we explored the possibility of simultaneously inactivating all the kinases. Since knocking down all the kinases involved with hairpins is technically cumbersome, we undertook a dominant-negative approach by stably transfecting MT4 cells with a kinase-inactive TBK1-K38A mutant with the rationale that this would block all endogenous kinases from phosphorylating CYLD. MT4 cells stably transfected with TBK1-K38A had reduced CYLD phosphorylation (Fig. 3a) and a significantly slower growth rate (Fig. 3b) compared to cells transfected with a control gene. Thus, both the inhibitor and dominant-negative studies showed a correlation between reduction of CYLD phosphorylation and reduced cellular viability of MT4 cells.

### IKK inhibitors induce CYLD-dependent cell death in MT4 cells

**Fig. 4:**
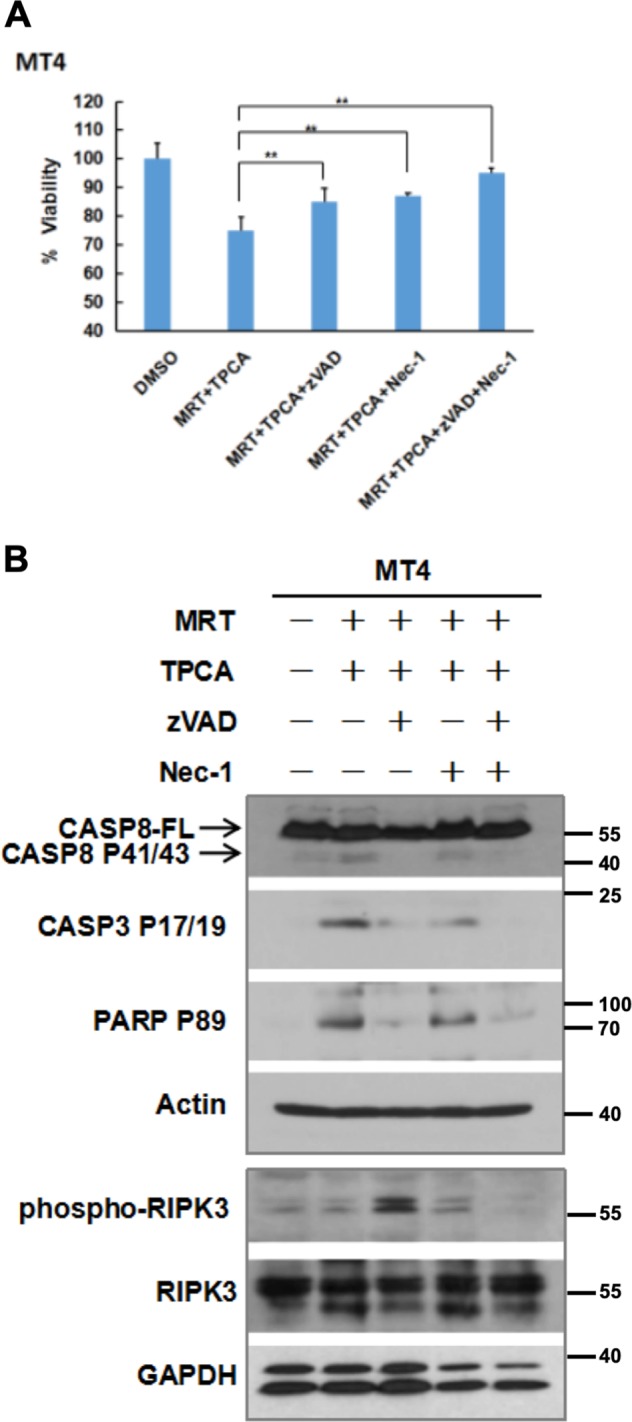
Inhibiting CYLD phosphorylation activates RIPK1-dependent apoptosis and necroptosis. **a** MT4 cells were treated for 24 h with a combination of MRT67307 (10 µM), TPCA (10 µM), zVAD-FMK (10 µM), and necrostatin-1 (30 µM) as indicated. Cellular viability was analyzed using CellTiter-Glo. The viability of cells treated with DMSO was set at 100%. The bars represent the mean ± S.D. from three independent experiments. *p < 0.05; **p < 0.01. **b** MT4 cells were treated as in (**a**). Lysates were sequentially blotted for Procaspase-8, cleaved Caspase-3, cleaved PARP-1, and β-actin. A separate membrane with identical samples was also blotted for phospho-RIPK3, RIPK3, and GAPDH. Cleaved Caspase-3 and PARP-1 are biochemical signatures for apoptosis. Phospho-RIPK3 is a biochemical signature for necroptosis.

We next examined the death pathway that is induced in MT4 cells treated with the combination of MRT67307 and TPCA. Cell death was partially blocked by the pan caspase inhibitor zVAD-FMK indicating that the cells are dying by apoptosis but could potentially switch to necroptosis when caspases are blocked (Fig. 4a). Blotting to detect cleavage of Caspase-8, Caspase-3, and PARP, which are biochemical hallmarks of apoptosis, confirmed this pathway is activated in the presence of the two IKK inhibitors (Fig. 4b). Presence of zVAD-FMK blocked these apoptotic markers but induced phosphorylation of RIPK3 (serine 227), indicative of a switch to necroptosis. Addition of necrostatin-1 to block RIPK1 kinase also partially inhibited cell death induced by the two IKK inhibitors (Fig. 4a) with a modest effect on Caspase-8, Caspase-3, and PARP cleavage (Fig. 4b). The combination of zVAD-FMK and necrostatin-1 was highly effective in blocking cell death. These results suggested that treatment with IKK inhibitors activated RIPK1-dependent cell death.

**Fig. 5:**
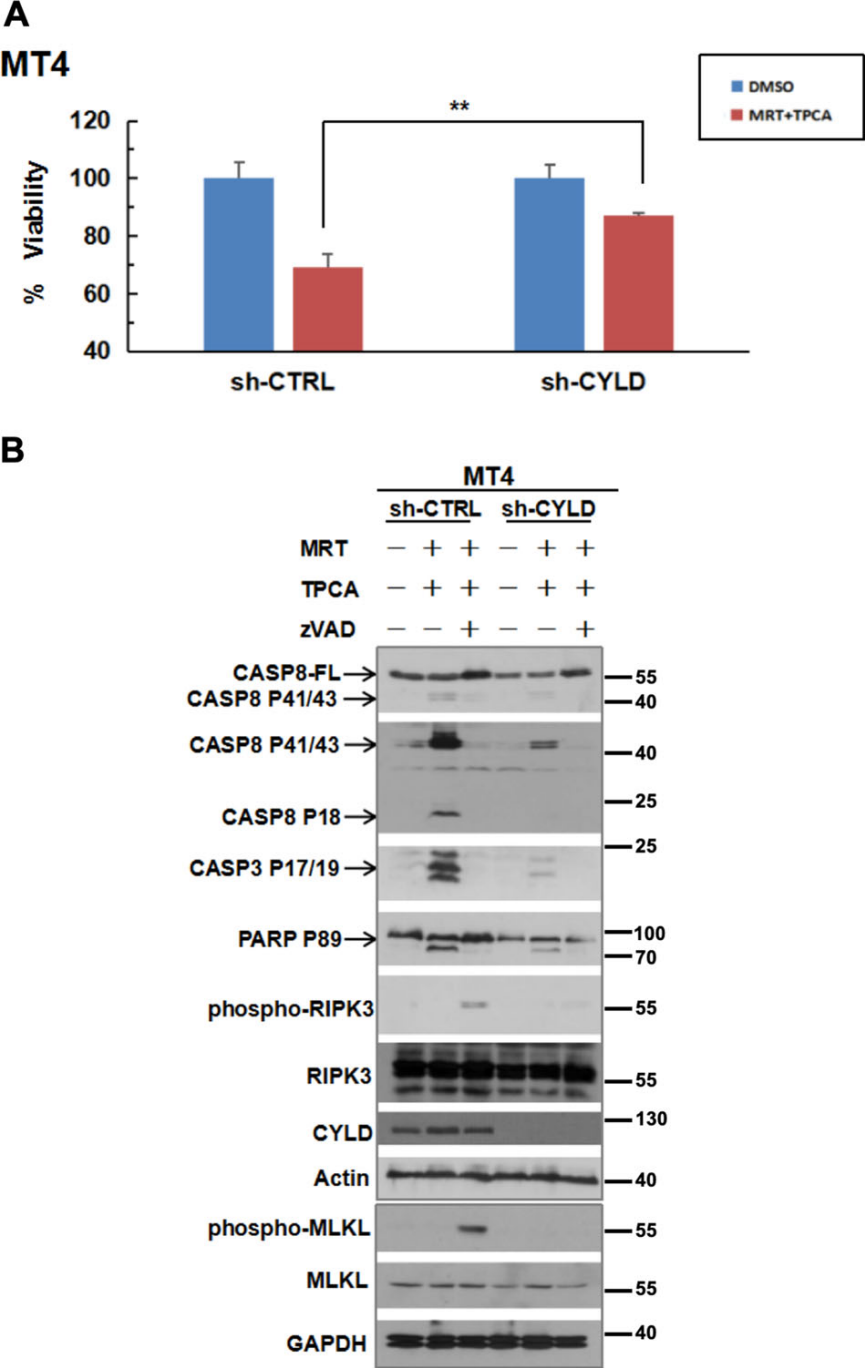
Loss of CYLD leads to decreased efficacy of IKK inhibitors in inducing apoptosis and necroptosis in MT4 cells. **a** MT4 cells were transduced with lentiviruses encoding a non-targeting or CYLD-targeting shRNA. After selection for stable knockdown of CYLD, the two lines were treated with a combination of 10 µM MRT67307 and TPCA for 24 h. Cell viability was assayed by CellTiter-Glo. The ATP level for each cell line treated with DMSO was set at 100%. The bars represent the mean ± S.D. from three independent experiments. **p < 0.01. **b** Control or CYLD-deficient cells were treated for 24 h with a combination of the kinase inhibitors in the absence or presence of zVAD-FMK (10 µM). Lysates were sequentially blotted for Procaspase-8, cleaved Caspase-8, cleaved Caspase-3, cleaved PARP-1, phospho-RIPK3, RIPK3, CYLD, and β-actin. A separate membrane with identical samples was also blotted for phospho-MLKL, MLKL, and GAPDH. Cleaved Caspase-3 and PARP-1 are biochemical signatures for apoptosis. Phospho-RIPK3 and phospho-MLKL are biochemical signatures for necroptosis.

**Fig. 6:**
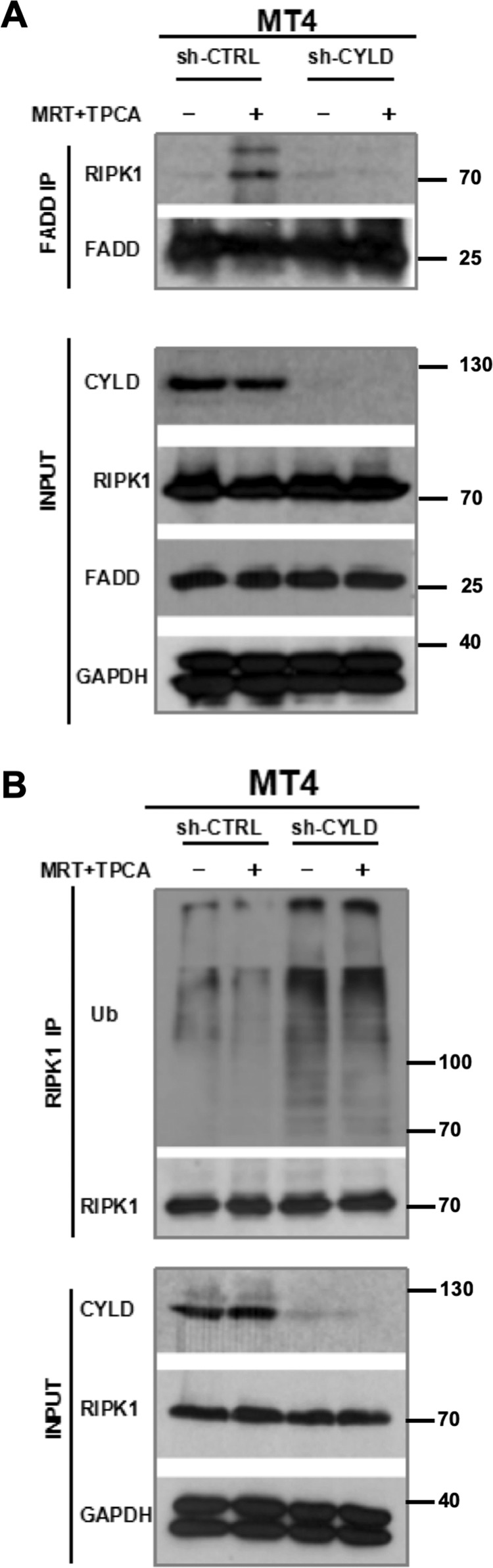
CYLD is crucial for RIPK1-FADD interaction and RIPK1 deubiquitination in MT4 cells. **a** Control or CYLD-deficient MT4 cells were treated for 16 h with a combination of MRT67307 and TPCA. Lysates were immunoprecipitated with anti-FADD and then sequentially blotted with anti-RIPK1 and anti-FADD. Equivalent portions of the lysates were also blotted as indicated in the lower panel. **b** Lysates from control or CYLD-deficient MT4 cells treated with kinase inhibitors for 8 h were immunoprecipitated with anti-RIPK1. Immune complex proteins were sequentially blotted with anti-ubiquitin and anti-RIPK1. Equivalent portions of the lysates were also blotted as indicated in the lower panel.

CYLD was shown to be an essential mediator in several studies on RIPK1-dependent apoptosis or necroptosis induced by TNF^25,32,45,46^. These studies demonstrated that CYLD is needed to remove ubiquitin from RIPK1 and switch it from a prosurvival signal to become a death-signaling molecule. Based on these prior reports and the data obtained thus far with the MT4 cells, we hypothesized that TAX activation of IKK family of kinases induces the phosphorylation of CYLD to suppress its deubiquitinating activity. We further hypothesized that this sustains RIPK1 ubiquitination and prevents RIPK1 from associating with death-signaling molecules. However, when CYLD phosphorylation is suppressed by IKK inhibitors, its enzymatic activity is then turned on to remove ubiquitin from RIPK1, allowing its association with death-signaling molecules. If this hypothesis was correct, cell death induced by the IKK inhibitors would be predicted to be dependent on CYLD. Consistent with this hypothesis, knockdown of CYLD in MT4 cells reduced the level of cell death following treatment with IKK inhibitors (Fig. 5a). Furthermore, both apoptosis (detected by Caspase-8, Caspase-3, and PARP cleavage) and necroptosis (detected by phospho-RIPK3 and phospho-MLKL) were diminished in CYLD-deficient cells treated with the IKK inhibitors (Fig. 5b). Association of RIPK1 with the FADD-containing death-inducing signaling complex (DISC) is a biochemical signature of the switching of RIPK1 to become a death-inducing molecule. Consistent with our hypothesis, IKK inhibitors induced the association of RIPK1 with the FADD DISC and this was dependent on CYLD (Fig. 6a). To confirm that IKK blockade leads to the induction of CYLD enzymatic activity, we examined the level of ubiquitination on its substrate RIPK1. As shown in Fig. 6b, IKK inhibitors reduced RIPK1 ubiquitination in control knockdown MT4 cells. More strikingly, RIPK1 ubiquitination was basally elevated in CYLD-deficient MT4 cells and it remained elevated upon IKK blockade. Our observations suggest that in cells transformed by HTLV-1, TAX induces the phosphorylation of CYLD to keep it inactive in order to prevent RIPK1 from inducing cell death. However, IKK blockade leads to the reactivation of CYLD, which then deubiquitinates RIPK1 enabling its association with the DISC.

### SMAC mimetic induces CYLD-dependent cell death in MT4 cells

**Fig. 7:**
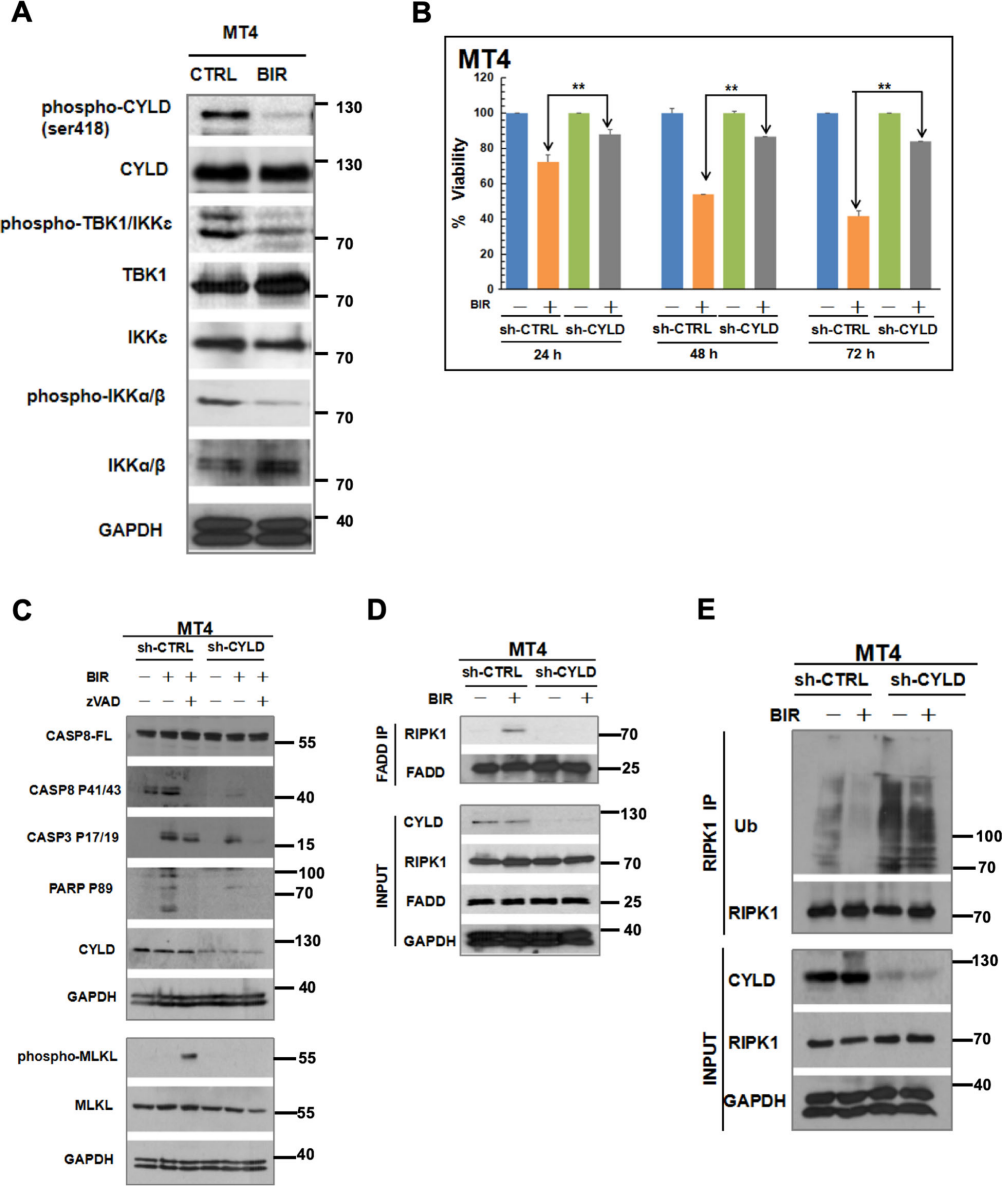
SMAC mimetic inhibits CYLD phosphorylation and kills MT4 cells in a CYLD-dependent manner. **a** MT4 cells were treated with media or 100 µM birinapant for 24 h. Lysates were sequentially blotted with the indicated antibodies. **b** Control or CYLD-deficient MT4 cells were treated with media or 100 µM birinapant for 24–72 h. Cell viability was assayed by CellTiter-Glo. The ATP level for each cell line treated with media at each time point was set at 100%. The bars represent the mean ± S.D. from three independent experiments. **p < 0.01. **c** Control or CYLD-deficient cells MT4 cells were treated for 24 h with 100 µM birinapant in the absence or presence of zVAD-FMK (10 µM). Lysates were sequentially blotted for Procaspase-8, cleaved Caspase-8, cleaved Caspase-3, cleaved PARP-1, CYLD, and GAPDH. A separate membrane with identical samples was also blotted for phospho-MLKL, MLKL, and GAPDH. **d** Control or CYLD-deficient MT4 cells were treated for 16 h with 100 µM birinapant. Lysates were immunoprecipitated with anti-FADD and then sequentially blotted with anti-RIPK1 and anti-FADD. Equivalent portions of the lysates were also blotted as indicated in the lower panel. **e** Lysates from control or CYLD-deficient MT4 cells treated with 100 µM birinapant for 8 h were immunoprecipitated with anti-RIPK1. Immune complex proteins were sequentially blotted with anti-ubiquitin and anti-RIPK1. Equivalent portions of the lysates were also blotted as indicated in the lower panel.

SMAC mimetics are currently being tested in a number of malignancies including those of the hematopoietic system^57,58^. These compounds degrade cIAP1 and cIAP2, the E3 ligases that mediate K63-linked ubiquitination of RIPK1^30–32^, and therefore disrupt the early cell death checkpoint. Thus, SMAC mimetics directly prevent RIPK1 ubiquitination and switch it to become a death-signaling molecule. Disruption in RIPK1 ubiquitination is also known to disrupt IKK activity^28,59^, and therefore we hypothesized that SMAC mimetics could also reduce IKK activity and CYLD phosphorylation. The resulting CYLD activation would result in further deubiquitination of RIPK1 and reduction in IKK activity. This could initiate a feed-forward loop that ultimately deubiquitinates RIPK1 sufficiently to enable it to associate with the DISC. We examined CYLD phosphorylation in MT4 cells after treating them with the SMAC mimetic birinapant (TL32711). Birinapant reduced CYLD phosphorylation as well as TBK1/IKKε and IKKα/β phosphorylation (Fig. 7a) and induced cell death in the MT4 cells, which was dependent on CYLD (Fig. 7b). Blotting indicated that birinapant induced apoptosis in MT4 cells, or necroptosis in the presence of zVAD-FMK, in a CYLD-dependent manner (Fig. 7c). Furthermore, birinapant induced the association of RIPK1 with the FADD-containing DISC in control but not in CYLD-knockdown MT4 cells (Fig. 7d). Consistent with our proposed mechanism, the ability of birinapant to reduce RIPK1 ubiquitination in MT4 cells was also dependent on CYLD expression (Fig. 7e). Similar effects were observed in MT4 cells treated with another SMAC mimetic LCL-161 (Supplementary Fig. 3).

## Discussion

Aggressive subtypes of ATLL have dismal prognoses and the only treatment with a potential for long-term remission is allogeneic stem cell transplant^14,16^. Hence, identifying signaling mechanisms critical for survival of ATLL cells is crucial for understanding mechanisms of resistance to current treatments and discovery of novel therapeutic targets for clinical trials.

CYLD was originally identified as a tumor suppressor gene that was mutated in familial cylindromatosis, Brooke–Spiegler syndrome and familial tricoepitheliomas^44^, which are autosomal dominant disorders predisposing to benign tumors of skin appendages. Subsequent studies have linked the loss of CYLD to the pathogenesis of several other tumors, including melanoma, breast cancer, T-lymphoblastic leukemia, and colon and hepatocellular carcinoma^48,60–62^. However, the role and regulation of CYLD in most hematological cancers, including ATLL, has not been well studied. Initial studies characterized CYLD as a key regulator of the NFκB pathway and because of the well-known role of NFκB in inducing prosurvival genes, its tumor suppressor function was largely thought to be via suppression of NFκB^44^. Based on our prior studies on how the early cell death checkpoint in the TNFR1 pathway is regulated, where we found that CYLD switches RIPK1 to become a death-signaling molecule^25,46,47^, we considered the possibility that the tumor suppressive function of CYLD might be due to its ability to switch RIPK1 to a death-signaling mode. Therefore in tumor cells, there would either be biallelic genetic loss of CYLD or posttranslational mechanisms to suppress CYLD. While there are some reports of genetic loss of CYLD in cancer cells^51,63–65^, this is not very common, suggesting the existence of posttranslational mechanisms that inhibit CYLD function. We had previously reported that CYLD could be proteolytically cleaved at aspartate 215, leading to its destruction^25,46,47^. Thus, we initially attempted to look for a signature of this cleavage event in transformed cells, without success.

While IKKα and IKKβ were initially isolated as kinases that phosphorylate IκBα, they have also been documented to phosphorylate substrates other than IκBs^1,66,67^. TBK1 and IKKε are known to phosphorylate IRF3/7 and STAT1 to regulate the type I interferon-signaling pathway. Seminal work by Sun and colleagues showed that CYLD was phosphorylated by IKKβ and this inhibited its catalytic activity^49^. Hutti et al. have reported that IKKε could also phosphorylate CYLD to inhibit its activity^48^. We had also observed that co-expression of either TBK1 or IKKε resulted in a mobility shift of CYLD indicative of phosphorylation^53^. These reports led us to consider phosphorylation of CYLD by both subfamilies of IKK as the mechanism by which it is suppressed in cancer cells. We tested this hypothesis in lymphomas because of our prior analysis showing that *CYLD*, *IKBKB* (encoding IKKβ), and *IKBKG* (encoding NEMO) are highly co-expressed in hemato-lymphoid cells^25,47^. This was indeed the case as we found CYLD to be highly phosphorylated in ATLL, together with elevated phosphorylation of IKKα/β and TBK1/IKKε, suggesting that CYLD may be suppressed in this manner in human ATLL. In addition to CYLD, these kinases can also directly phosphorylate RIPK1 to inhibit cell death. IKKβ, TBK1, and IKKε have all been reported to phosphorylate RIPK1 to suppress the death-signaling function of RIPK1^33–37^. It is likely that the IKK family phosphorylates multiple targets including RIPK1 and CYLD, all with the goal of inhibiting RIPK1-mediated cell death.

**Fig. 8:**
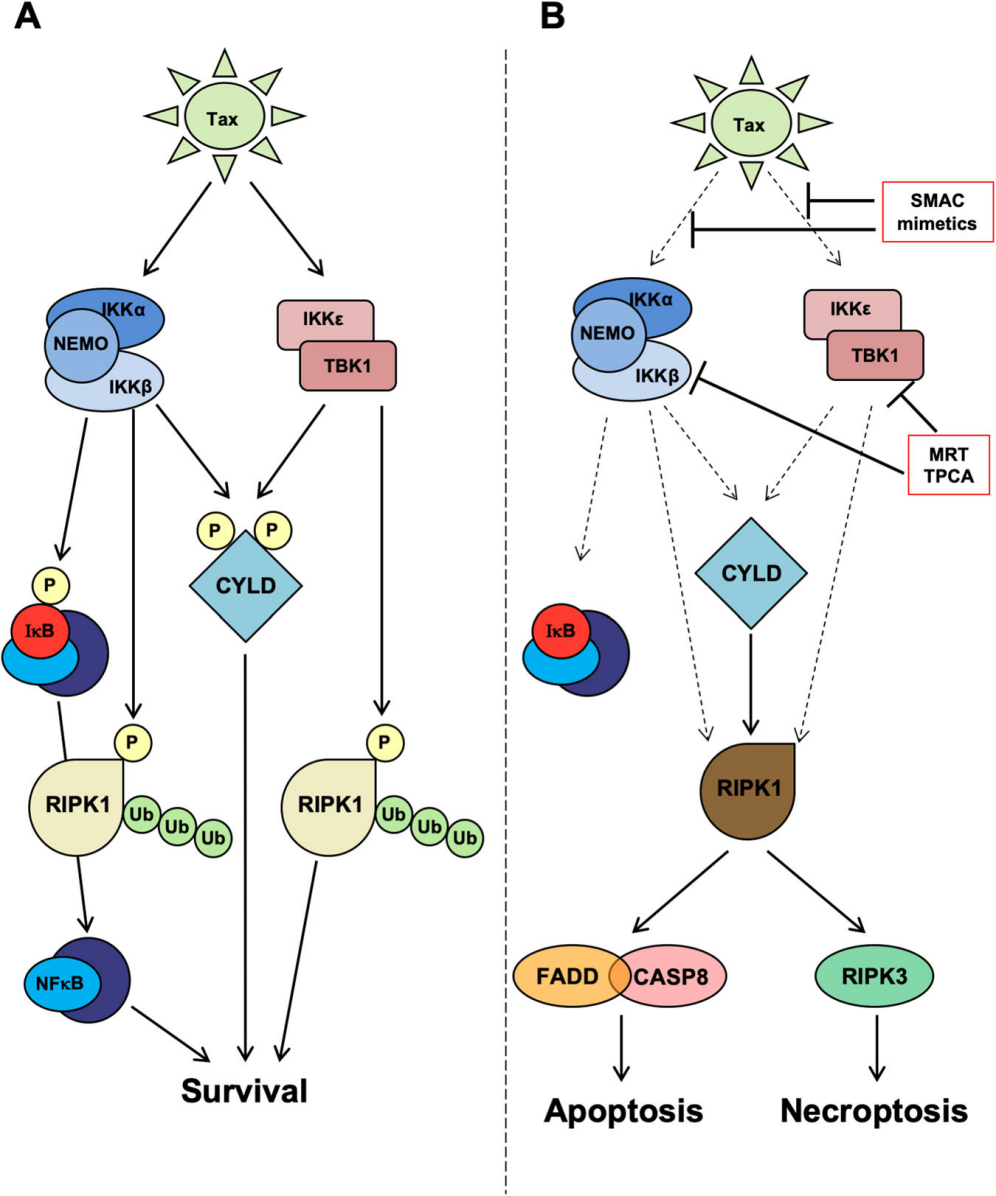
Proposed model of TAX medicated inactivation of CYLD resulting in suppression of RIPK1-dependent cell death in ATLL. **a** Viral oncogene TAX maintains the early cell death checkpoint by driving multiple IKK to phosphorylate and suppress CYLD. This inhibits the removal of ubiquitin chains from RIPK1 thereby preventing its association with downstream death-signaling molecules. The IKKs also directly phosphorylate RIPK1 to inhibit its death-signaling activity. The IKKs also activate NFκB transcription factors via phosphorylation of IκB proteins to induce a transcription-dependent survival program. **b** Disruption of this early cell death checkpoint leads to CYLD- and RIPK1-dependent death. This can be achieved by blocking the ubiquitin scaffold required for IKK activity, or directly inhibiting IKK enzymatic activity, both of which impair CYLD and RIPK1 phosphorylation. Reduced phosphorylation reactivates CYLD to remove ubiquitin chains from RIPK1, which together with impaired phosphorylation of RIPK1, enables RIPK1 to now associate with death-signaling molecules to initiate apoptosis or necroptosis in ATLL cells. Death is further facilitated by impaired expression of NFκB-dependent survival genes.

To understand further the role that CYLD phosphorylation may be playing in ATLL pathogenesis, we analyzed this modification in HTLV-1 transformed T-cell lines, representative of ATLL, as well as in primary human ATLL samples. In both of these, there was constitutive phosphorylation of CYLD, accompanied by phosphorylation of IKKα/β and TBK1/IKKε, indicative of active kinases. To gain mechanistic insights, we studied lymphoma models driven by the retroviral oncogene TAX, a known activator of IKKs. In these models, inhibition of TBK1/IKKε using MRT67307 and IKKβ using TPCA showed that both subfamilies could phosphorylate CYLD, with the combination of the two inhibitors having a marked effect in reducing phosphorylation and triggering cell death. Because it was technically challenging to knockdown all four kinases of the IKK family simultaneously in a stable format, we resorted to using a dominant negative approach overexpressing a kinase-inactive TBK1, which could potentially block all the CYLD kinases. TBK1-K38A expression blocked CYLD phosphorylation and inhibited the proliferation of MT4 cells. IKK blockade reactivated CYLD, as evidenced by the reduction in RIPK1 ubiquitination, which led to the association of RIPK1 with the DISC, triggering cell death. In the absence of CYLD, RIPK1 ubiquitination remained elevated following IKK blockade and it did not associate with the DISC. These observations strongly suggest that in ATLL cells, either viral encoded oncogenic proteins or mutations sustain the early cell death checkpoint by driving multiple IKKs to phosphorylate and suppress CYLD in order to prevent RIPK1 from becoming a death-signaling molecule (Fig. 8a). Conversely, disrupting CYLD phosphorylation using IKK inhibitors reactivates CYLD, which in turn, deubiquitinates and switches RIPK1 into a death-signaling molecule (Fig. 8b). Apoptosis appears to be the dominant type of cell death induced following reactivation of CYLD in the lymphoma cell lines examined. This was inferred from the apoptotic signature of Caspase-3 and PARP cleavage and the lack of the necroptotic signature of phospho-RIPK3 or phospho-MLKL when cells were treated with only IKK inhibitors. However, consistent with this being RIPK1-mediated death, caspase inhibition could switch the death response to necroptosis.

The early cell death checkpoint is highly amenable to pharmacologic modulation, since it is regulated by a network of enzymes. We provide evidence to show that SMAC mimetics, which are being evaluated as antitumor agents, function similarly to the IKK inhibitors to disrupt CYLD phosphorylation. SMAC mimetics degrade cIAP1/2, the E3 ligases that ubiquitinate RIPK1^30,32^. It is widely assumed that in the presence of SMAC mimetics, the failure to ubiquitinate RIPK1 is sufficient to turn it into a death-signaling molecule. The fact that SMAC mimetics required CYLD to kill cells in response to TNF, as was reported previously^32^ and also in the current study, indicates that the picture may be more complicated. In CYLD-depleted cells, birinapant and LCL-161 were not able to reduce RIPK1 ubiquitination. The requirement for CYLD means that it has to be activated following SMAC mimetic treatment. We propose that initial inhibition of K63-linked ubiquitination on RIPK1 brought about by SMAC mimetics leads to a reduction in IKK activity (Fig. 8b). This reduces CYLD phosphorylation, activating this enzyme to remove more ubiquitin molecules from RIPK1, thus setting up a positive-feedback loop to deubiquitinate RIPK1 to the level allowing its association with the DISC.

While posttranslational mechanisms that prevent CYLD and RIPK1 from becoming death-signaling molecules in the early death checkpoint are crucial to lymphoma cell survival, the induction of NFκB-dependent prosurvival genes in the late checkpoint is also integral and this has been widely studied. The two checkpoints are functionally linked in that components of the machinery involved in the early checkpoint (e.g., IKK) eventually also activate NFκB in the late checkpoint. The IKKβ inhibitor TPCA that was used in this study is known to inhibit NFκB gene induction so the death caused by the combination of MRT67307 and TPCA is due in part to the loss of NFκB-dependent gene transcription of prosurvival genes. Similarly, CYLD is also known to inhibit NFκB signaling and therefore, inhibitory phosphorylation of CYLD will also serve to enhance expression of prosurvival genes. However, the two checkpoints perform very different functions and it would be advantageous for the malignant cell to reinforce both checkpoints. The early checkpoint serves to specifically inhibit RIPK1 from becoming a death-signaling molecule whereas the late checkpoint serves to maintain a global state of death resistance. Indeed, TAX is known to be a strong activator of NFκB^68^ due in part to TAX interaction with NEMO and activation of IKK activity^69–71^. Our results now suggest that TAX also reinforces the early cell death checkpoint to suppress RIPK1’s death-signaling function. Nonetheless, it is possible for death-signaling CYLD and RIPK1 molecules, activated by disruption of the early checkpoint, to override the protection provided by the second NFκB-dependent checkpoint. This is illustrated by SMAC mimetics, which activate the noncanonical NFκB signaling pathway^72,73^. Despite the induction of noncanonical NFκB signaling, which is highly prosurvival^74^, the cells remain vulnerable to CYLD and RIPK1-dependent death in the presence of SMAC mimetics and TNF.

While there are limitations with the pharmacological and dominant-negative approaches used in this study, nonetheless the observations strongly suggest that phosphorylation of CYLD is a mechanism used by ATLL cells to suppress this tumor suppressor and maintain tumor cell survival. Disruption of this modification leads to the reactivation of CYLD and one consequence of that is initiation of the RIPK1-dependent cell death pathway. Our findings support strategies inhibiting CYLD phosphorylation as novel therapeutic approaches for ATLL patients.

## Materials and methods

### Human ATLL samples

The primary human ATLL samples used for analysis were left-over, de-identified and anonymized specimens obtained from adults at the time of diagnosis, prior to any therapy, according to protocols approved by the institutional review boards of Mount Sinai Medical Center and Columbia University Medical Center. Informed consent was obtained from all subjects. ATLL was diagnosed using the WHO 2017 criteria^75^, based on morphologic features, immunophenotype, and clinical presentation. ATLL samples were frozen in optimal cutting temperature (OCT) compound. The tissue was thawed on ice and washed once with cold phosphate-buffered saline by centrifugation at 1000 rpm on a table top centrifuge for 15 min. After discarding the supernatant, the tissue was lysed with Triton lysis buffer for 20 min on ice. The lysates were sonicated with six 10-s bursts with a probe sonicator on ice. Lysates were microfuged at 14,000 rpm for 15 min and an aliquot of the supernatants removed for BCA assay to determine protein concentrations prior to SDS-PAGE.

### Cell lines

The Jurkat T-cell subclone 3T8 was cultured as previously described^52^. The HEK 293 derivative cell line 293EBNA was obtained from Invitrogen and cultured as described^76^. HUT 78, C8166, MT4, MT2, and SLB1 T lymphoma cell lines were obtained from the NIH AIDS Reagent Program and kindly provided by Dr. Benjamin Chen (Icahn School of Medicine at Mount Sinai, NY) and Dr. Owen A. O’Connor (Columbia University Medical Center, NY). All cell lines were frequently checked for their morphological features and functionality. All cells were grown at 37 °C in a 5% CO_2_ incubator. HUT 78, C8166, MT4, MT2, and SLB1 are cultured in RPMI medium with 10% fetal bovine serum and pen–strep.

### Antibodies

Antibodies were purchased from the indicated vendors. GAPDH (clone D-6) and TAX (clone 1A3) were from Santa Cruz Biotechnology. Phospho-CYLD(Ser418) (#4500), CYLD (clone D1A10), Phospho-TBK1/NAK (Ser172) (clone D52C2), TBK1/NAK (clone D1B4), IKKɛ (#2690), Phospho-IKKɑ/β (Ser176/180) (clone 16A6), IKKβ (clone D30C6), IKKɑ (#2682), cleaved Caspase-8 (Asp391) (clone 18C8), Caspase-8 (clone 1C12), cleaved Caspase-3 (Asp175) (clone 5A1E), cleaved PARP-1 (Asp214) (#9541), ubiquitin (clone P4D1), and β-actin (clone 8H10D10) were from Cell Signaling Technology. Phospho-MLKL (Ser358) (clone EPR9514) and phospho-RIPK3 (Ser227) (clone EPR9627) were obtained from Abcam. RIPK1 (clone 38/RIP) and FADD (clone A66-2) were obtained from BD Biosciences. MLKL (clone 3H1) and FADD (#06-711) were obtained from Millipore Corp. RIPK3 (#2283) was obtained from ProSci. FLAG (clone M2) was obtained from Sigma.

### Reagents

Birinapant and LCL-161 were purchased from MedChemExpress. zVAD-fmk was purchased from Bachem. Necrostatin-1 was obtained from Tocris Bioscience. MRT67307, TPCA-1 [5-(p-Fluorophenyl)-2-ureido]thiophene-3-carboxamide and Protease Inhibitor Cocktail Set V were purchased from EMD Millipore.

### Plasmids

pCMV4-TAX WT was a gift from Dr. Warner Greene (Addgene plasmid #23284). The TBK1-K38A ORF was amplified by polymerase chain reaction from a template provided by Dr. Benjamin tenOever (Icahn School of Medicine at Mount Sinai, NY) and subcloned into an Moloney murine leukemia virus-based retroviral vector upstream of an internal ribosome entry site-puromycin resistance cassette. The negative control retroviral vector encodes bacterial GST. A non-targeting shRNA or CYLD-targeting shRNA (SHCLNG-NM_015247, TRCN0000039629) encoded by the lentiviral vector pLKO.1-puro were obtained from Sigma-Aldrich.

### Transfection

TAX and myc-CYLD expression plasmids were transfected into HEK 293EBNA cells by calcium phosphate precipitation. Retroviral transduction of lymphoma cells was performed as previously described^77^. Lentivirus particles encoding shRNA were pseudotyped and packaged in a similar manner as for retroviruses. Forty-eight hour after transduction, lymphoma cells were stably selected using puromycin.

### Immunoblotting

For western blotting, 1–2 × 10^6^ cells in each sample were lysed in buffer containing 1% Triton X-100 as previously described^46^. For each sample, 30–70 μg of protein was resolved by 8, 10, or 12% SDS-PAGE and transferred to nitrocellulose membranes. Protein detection was carried out by incubating membranes with primary antibodies overnight at 4 °C, followed by incubating membranes with secondary antibody for 1 h at room temperature. Blot visualization was carried out using chemiluminescence (ECL reagent). Western blot analysis of lysates from cell lines were performed three times. The western blot analysis of lysates from human ATLL specimens was performed once.

### ATP viability assay

Cells were seeded 2.5 × 10^4^ cells/well in 96-well plates. After treatment, viability was analyzed using the CellTiter-Glo^®^ Luminescent Cell Viability Assay kit (Promega) according to the manufacturer’s instruction.

### Immunoprecipitation

In all, 10 × 10^6^ cells per sample were treated and then lysed in buffer containing 20 mM Tris pH 7.4, 150 mM sodium chloride, 10% glycerol, 0.2% NP-40, 0.1 mM sodium orthovandate, 5 mM glycerophosphate and Protease Inhibitor Cocktail Set V for 20 min on ice. Lysates were cleared by centrifugation at 10,000 × *g* at 4 °C and protein concentration measured using Pierce BCA (ThermoFisher Scientific). Equivalent amount of protein in each sample were immunoprecipitated by rotating with 0.25–1 µg of FADD antibody overnight at 4 °C. Immune complexes were precipitated with Protein A/G beads. After extensive washing, the beads were eluted with SDS-sample buffer at 70 °C for 20 min. Sequential blotting with anti-RIPK1 and anti-FADD was carried out subsequently. To detect RIPK1 ubiquitination, immunoprecipitation was carried out using anti-RIPK1 followed by sequential blotting with anti-ubiquitin and anti-RIPK1.

### Statistical analysis

Bar charts display mean values and the error bars indicate the S.D. of the sample sizes described in the figure legends. Two-tailed Student’s *t* test (assuming equal variance) was used to determine statistical significance and the p values are shown in the figure legends. (*p < 0.05, **p < 0.01)

## Supplementary information

Supplementary Figure 1

Supplementary Figure 2

Supplementary Figure 3

Supplementary Figure Legends

Author Contribution
