# Supplementary figures and images for: Reversal of CYLD phosphorylation as a novel therapeutic approach for adult T-cell leukemia/lymphoma (ATLL)
Source: Cell Death Dis. 2020 Feb 5;11(2):94. doi: 10.1038/s41419-020-2294-6 (PMC7002447; doi:10.1038/s41419-020-2294-6)

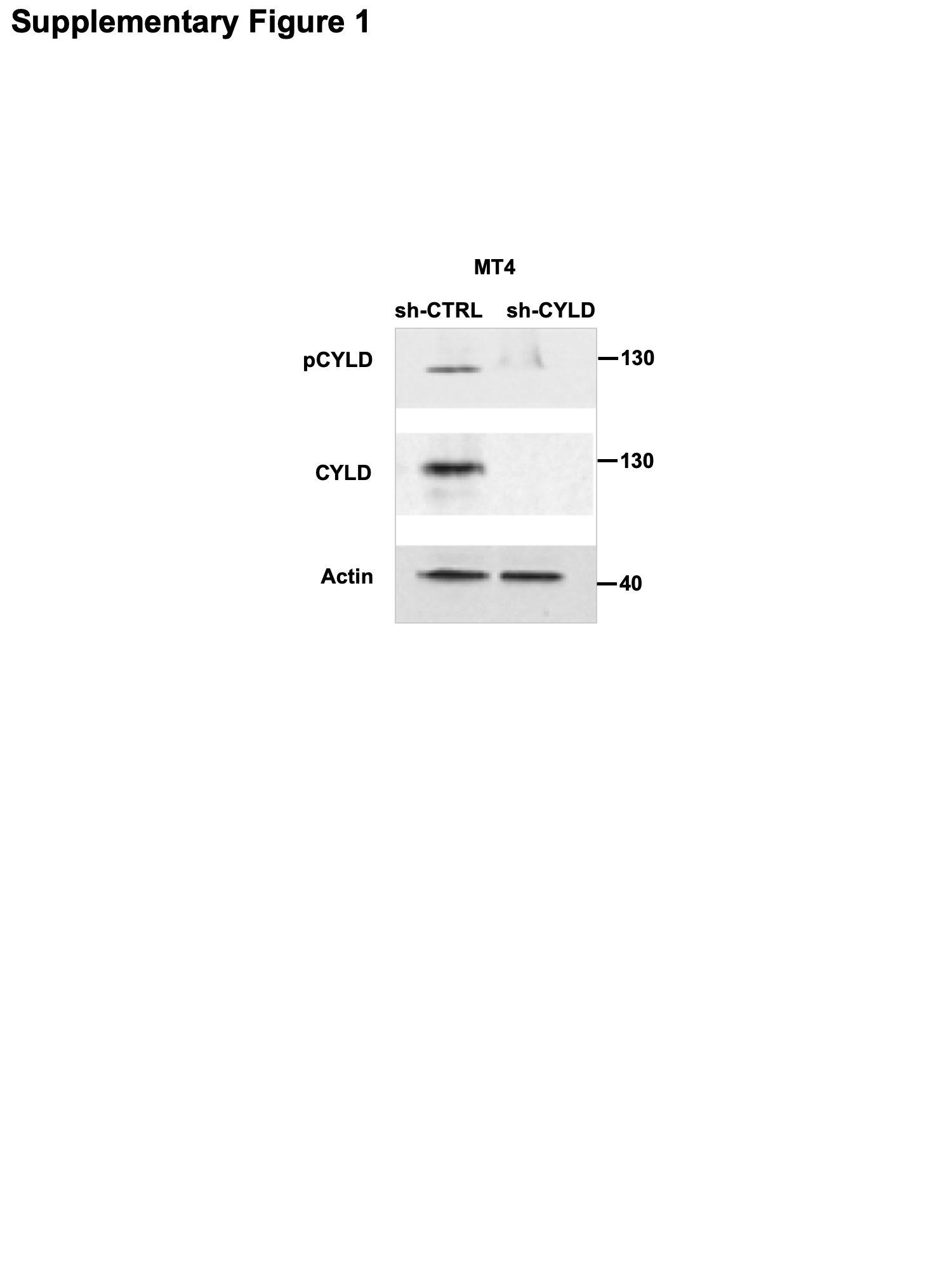

Supplement: Supplementary file 1 — Supplementary Figure 1 [file 41419_2020_2294_MOESM1_ESM.tif]

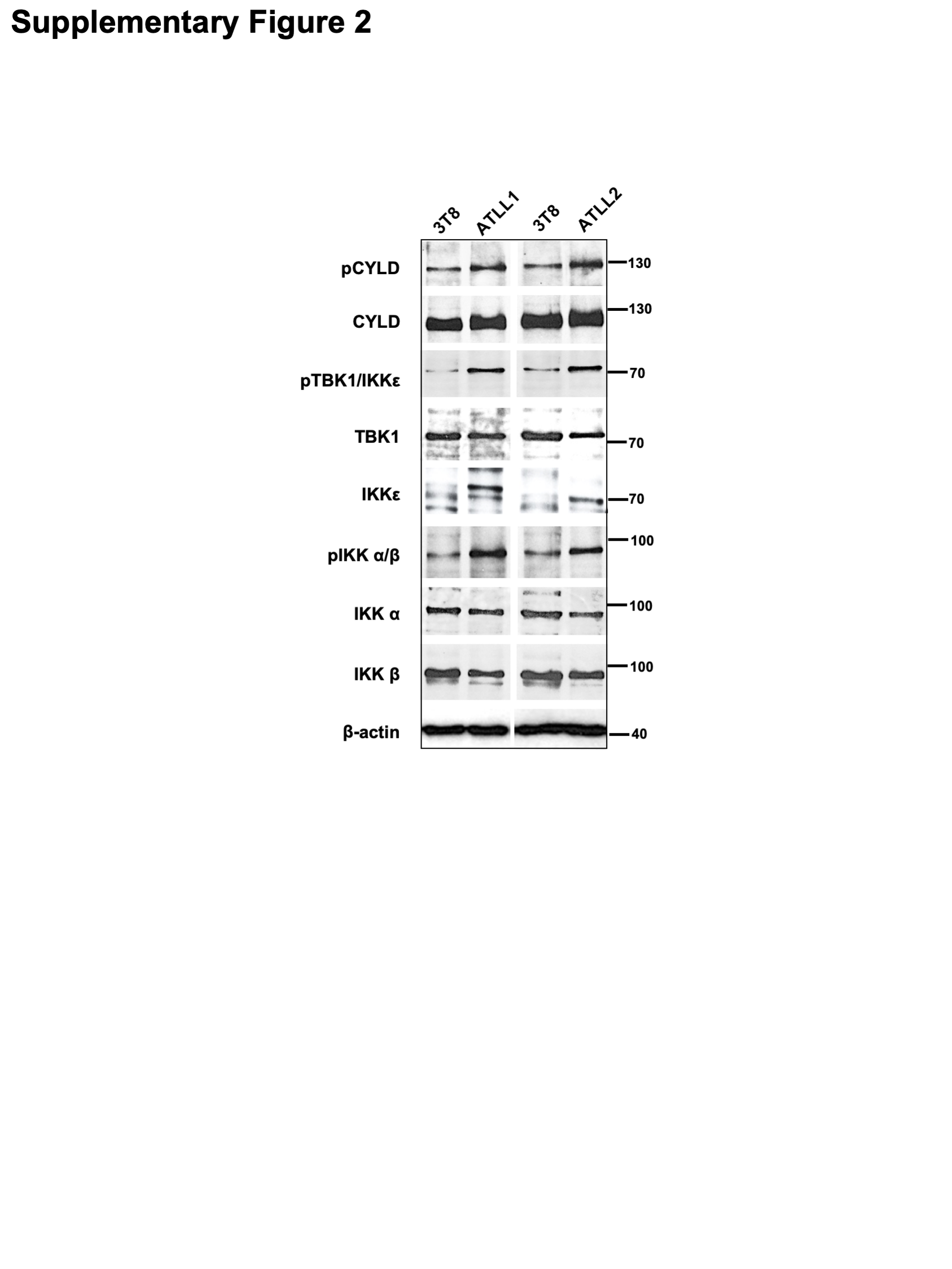

Supplement: Supplementary file 2 — Supplementary Figure 2 [file 41419_2020_2294_MOESM2_ESM.tif]

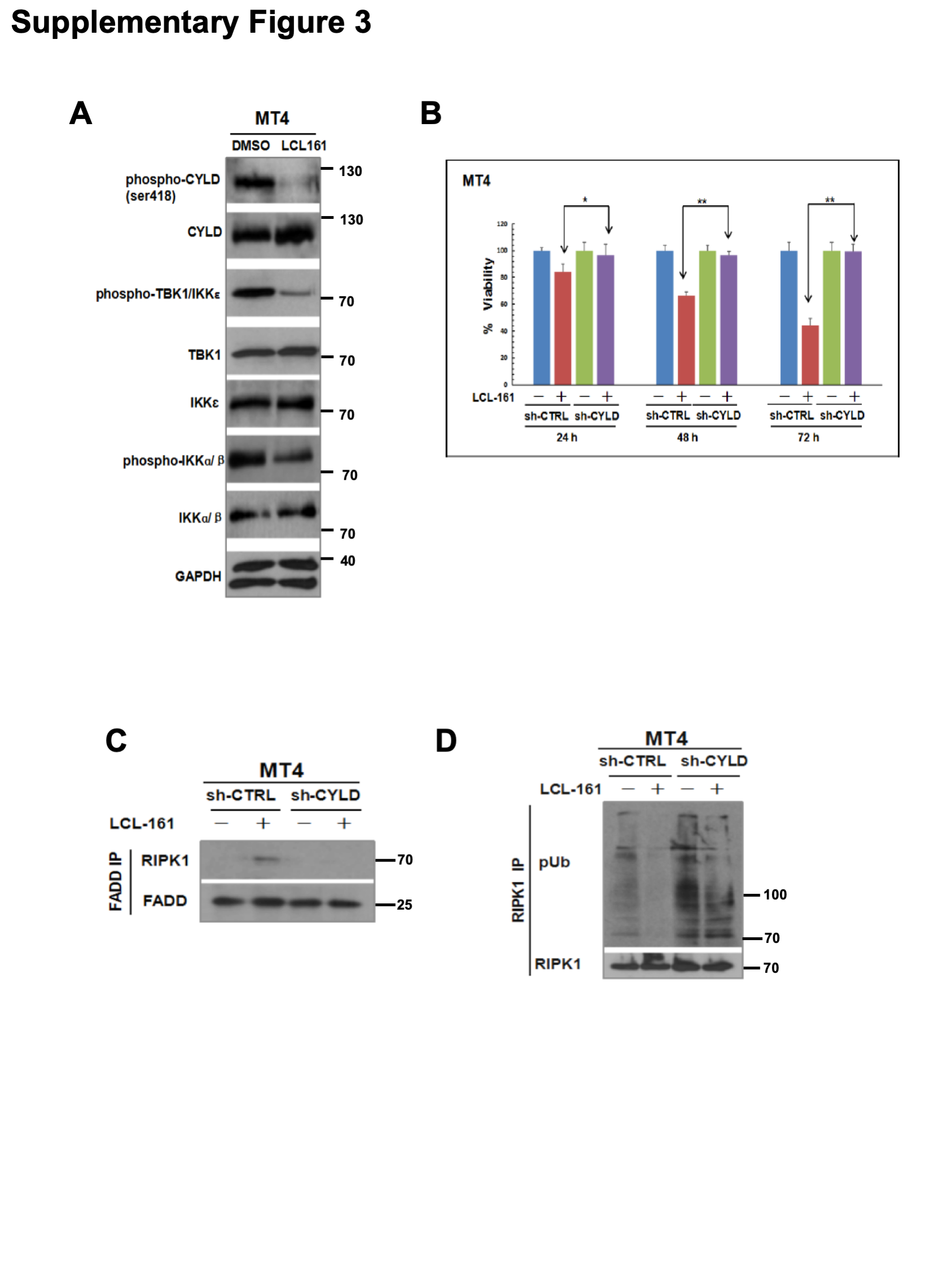

Supplement: Supplementary file 3 — Supplementary Figure 3 [file 41419_2020_2294_MOESM3_ESM.tif]
