# Supplementary material for: Reversal of CYLD phosphorylation as a novel therapeutic approach for adult T-cell leukemia/lymphoma (ATLL)
Source: Cell Death Dis. 2020 Feb 5;11(2):94. doi: 10.1038/s41419-020-2294-6 (PMC7002447; doi:10.1038/s41419-020-2294-6)
Supplement: Supplementary file 4 — Supplementary Figure Legends [file 41419_2020_2294_MOESM4_ESM.docx]

**Supplementary Figure Legends**

Supplementary Figure 1. Phospho-S418-CYLD antibody detects phosphorylated CYLD in MT4 cells.

(A) MT4 cells were transduced with lentiviruses encoding a non-targeting or CYLD-targeting shRNA. After selection for stable knockdown of CYLD, lysates from the two lines were sequentially probed for phospho-CYLD, total CYLD and beta actin as a loading control. The phospho-specific antibody detected a signal in the control MT4 cells but not in the CYLD-deficient MT4 cells indicating that the antibody is specific.

Supplementary Figure 2. Increased CYLD phosphorylation in tumor cells from ATLL patients.

Protein extracts from tumor cells obtained from two ATLL patients (lane 2 and 4) were blotted sequentially for phospho-CYLD, CYLD, phospho-TBK1/IKKε, TBK1, IKKε, phospho-IKKα/β, IKKα/β and beta actin as a loading control. Extracts from Jurkat T cells (clone 3T8) were used as controls (lane 1 and 3).

Supplementary Figure 3. SMAC mimetic LCL-161 inhibits CYLD phosphorylation and kills MT4 cells in a CYLD-dependent manner.

(A) MT4 cells were treated with media or 10μM of LCL-161 for 24h. Lysates were sequentially blotted with the indicated antibodies.

(B) Control or CYLD-deficient MT4 cells were treated with media or 10μM of LCL-161 for 24 to 72 h. Cell viability was assayed by CellTiter-Glo. The ATP level for each cell line treated with media at each time point was set at 100%. The bars represent the mean +/- S.D. from three independent experiments. **p < 0.01.

(C) Control or CYLD-deficient MT4 cells were treated for 16 h with 10 μM of LCL-161. Lysates were immunoprecipitated with anti-FADD and then sequentially blotted with anti-RIPK1 and anti-FADD.

(D) Lysates from control or CYLD-deficient MT4 cells treated with 10 µM LCL-161 for 8 h were immunoprecipitated with anti-RIPK1. Immune complex proteins were sequentially blotted with anti-ubiquitin and anti-RIPK1.
