# Supplementary material for: Reversal of CYLD phosphorylation as a novel therapeutic approach for adult T-cell leukemia/lymphoma (ATLL)
Source: Cell Death Dis. 2020 Feb 5;11(2):94. doi: 10.1038/s41419-020-2294-6 (PMC7002447; doi:10.1038/s41419-020-2294-6)
Supplement: Supplementary file 5 — Author Contribution [file 41419_2020_2294_MOESM5_ESM.pdf]

DECLARATION OF CONTRIBUTIONS TO ARTICLE

ADMC

Manuscript Number: CDDIS-19-2710

Journal Name: *Cell Death & Disease* (the 'Journal')

Proposed Title of the Contribution:

Reversal of CYLD phosphorylation as a novel therapeutic approach for adult T cell leukemia/lymphoma (ATLL) (the 'Contribution)

Author(s):

Xin Xu, Matko Kalac, Michael Markson, Mark Chan, Joshua D. Brody, Govind Bhagat, Rosalind L. Ang, Diana Legarda, Scott J. Justus, Feng Liu, Qingshan Li, Huabao Xiong, Adrian T. Ting (the 'Authors')

For all *CDDis* articles, each person named as an author in the published version must be able to show he or she has contributed substantially to the article.

Authorship credit should be based on 1) substantial contributions to conception and design, acquisition of data, or analysis and interpretation of data; 2) drafting the article or revising it critically for important intellectual content; and 3) final approval of the version to be published. Authors should meet conditions 1, 2 and 3.

Any person who cannot be shown to have made a substantial contribution to the article cannot be listed as an author in the final version. The name of any person who is deemed to have made a minor contribution can, however, appear in the Acknowledgments section of the article.

Please complete the table below to indicate the contributions of all named authors to the manuscript.

Author Full Name:	Specification of Contribution to the Manuscript:
Xin Xu	Design & oversaw the project, performed experiments, analyzed data and wrote the manuscript.
Matko Kalac	Performed experiments, analyzed data and wrote the manuscript.
Michael Markson	Performed experiments and collected data.
Mark Chan	Performed experiments and collected data.
Joshua D. Brody	Procured human tumor cells/tissues and provided critical comments.
Govind Bhagat	Procured human tumor cells/tissues and provided critical comments.
Rosalind L. Ang	Generated and validated reagents.
Diana Legarda	Generated and validated reagents.
Scott J. Justus	Generated and validated reagents.
Feng Liu	Provided fellowship funding support and critical comments.
Qingshan Li	Provided fellowship funding support and critical comments.
Huabao Xiong	Provided technical guidance and critical comments.
Adrian T. Ting	Design & oversaw the project, analyzed data and wrote the manuscript.

Please complete the table below to indicate the contributions of all named authors to the figures.

Figure 1:

X.X., M.K. and M.M. generated data and prepared panels.

Figure 2:

X.X. generated data and prepared panels.

Figure 3:

D.L. generated and validated reagents. X.X. generated data and prepared panels.

Figure 4:

X.X. generated data and prepared panels.

Figure 5:

D.L. and S.J.J. generated and validated reagents. X.X., M.K., M.C., and M.M. generated data and prepared panels.

Figure 6:

R.L.A validated reagents. X.X. generated data and prepared panels.

Figure 7:
X.X. generated data and prepared panels.

Signed for and on behalf of the Author(s):	Print Name:	Date:
[signature]	Adrian Ting	12/05/2019
